# Ganghuo Kanggan Decoction in Influenza: Integrating Network Pharmacology and *In Vivo* Pharmacological Evaluation

**DOI:** 10.3389/fphar.2020.607027

**Published:** 2020-12-10

**Authors:** Yanni Lai, Qiong Zhang, Haishan Long, Tiantian Han, Geng Li, Shaofeng Zhan, Yiwei Li, Zonghui Li, Yong Jiang, Xiaohong Liu

**Affiliations:** ^1^ The First Clinical Medical College, Guangzhou University of Chinese Medicine, Guangzhou, China; ^2^ Laboratory Animal Center, Guangzhou University of Chinese Medicine, Guangzhou, China; ^3^ The First Affiliated Hospital of Guangzhou University of Chinese Medicine, Guangzhou, China; ^4^ Shenzhen Hospital of Integrated Traditional Chinese and Western Medicine, Shenzhen, China

**Keywords:** ganghuo kanggan decoction, influenza, network pharmacology, RIG-I-like receptors signal pathway, pneumonia, type I interferon

## Abstract

**Background:** Ganghuo Kanggan decoction (GHKGD) is a clinical experience prescription used for the treatment of viral pneumonia in the Lingnan area of China, and its clinical effect is remarkable. However, the mechanism of GHKGD in influenza is still unclear.

**Objective:** To predict the active components and signaling pathway of GHKGD and to explore its therapeutic mechanism in influenza and to verified it *in vivo* using network pharmacology.

**Methods:** The potential active components and therapeutic targets of GHKGD in the treatment of influenza were hypothesized through a series of network pharmacological strategies, including compound screening, target prediction and pathway enrichment analysis. Based on the target network and enrichment results, a mouse model of influenza A virus (IAV) infection was established to evaluate the therapeutic effect of GHKGD on influenza and to verify the possible molecular mechanism predicted by network pharmacology.

**Results:** A total of 116 candidate active compounds and 17 potential targets were identified. The results of the potential target enrichment analysis suggested GHKGD may involve the RLR signaling pathway to reduce inflammation in the lungs. *In vivo* experiments showed that GHKGD had a protective effect on pneumonia caused by IAV-infected mice. Compared with the untreated group, the weight loss in the GHKGD group in the BALB/c mice decreased, and the inflammatory pathological changes in lung tissue were reduced (*p* < 0.05). The expression of NP protein and the virus titers in lung were significantly decreased (*p* < 0.05). The protein expression of RIG-I, NF-kB, and STAT1 and the level of MAVS and IRF3/7 mRNA were remarkably inhibited in GHKGD group (*p* < 0.05). After the treatment with GHKGD, the level of Th1 cytokines (IFN-γ, TNF-α, IL-2) was increased, while the expression of Th2 (IL-5, IL4) cytokines was reduced (*p* < 0.05).

**Conclusion:** Through a network pharmacology strategy and *in vivo* experiments, the multi-target and multi-component pharmacological characteristics of GHKGD in the treatment of influenza were revealed, and regulation of the RLR signaling pathway during the anti-influenza process was confirmed. This study provides a theoretical basis for the research and development of new drugs from GHKGD.

## Introduction

Influenza is an acute infectious respiratory disease caused by the influenza virus. It has high morbidity, strong infectivity, widespread epidemic potential and high mortality (Cordova-Villalobos et al., 2017). Influenza virus pandemics, which are also common causes of death, are mainly caused by IAV. In 2009, a new type of H1N1 influenza virus caused the “Mexican influenza pandemic,” which affected 214 countries and regions worldwide and caused more than 290,000 deaths, 201,000 of which were from respiratory failure (Buchholz et al., 2016). Death from severe influenza due to respiratory failure is closely related to increased permeability of the alveolar epithelial-vascular endothelial barrier and excessive release of inflammatory factors (Herold et al., 2015). Severe viral infections can induce the release of a large amount of cytokines in the body, causing a severe imbalance between the body’s pro-inflammatory and anti-inflammatory cytokines, inducing cytokine storm and targeting the lungs, thus triggering acute lung injury (ALI) and even acute respiratory distress syndrome (ARDS) (Chen et al., 2018). With the rapid mutation of influenza viruses and the emergence of resistance to antiviral drugs, current vaccines and drugs still cannot effectively control the widespread spread of influenza viruses. As a result, an increasing number of scientists are focusing on the development of drugs against host targets (Hsieh et al., 2018). Therefore, how to effectively control inflammation before the inflammatory factor storm occur is an effective means to reduce the progression of influenza to ALI or ARDS.

Innate immunity is the first line of defense against pathogen invasion. Retinoic acid-inducible gene I (RIG-I), one of the main members of the RIG-I-like receptor (RLRs) family, plays an important role in the identification of intracellular influenza viruses and triggering antiviral and inflammatory responses (Liu et al., 2019). After RIG-I recognizes the 5′-triphosphate influenza virus single-stranded RNA (ssRNA), its helicase domain binds to ATP to activate the mitochondrial antiviral signal protein. The interaction between mitochondrial antiviral signaling protein (MAVS) and the stimulator of interferon genes (STING) on the endoplasmic reticulum further activates NF-κB and interferon regulatory factor 3/7 (IRF3/7), which promotes the production of pro-inflammatory factors and type I and type III interferons (Liu et al., 2019). This causes the infected cells and surrounding tissues enter an antiviral state (Lin et al., 2019).

Traditional Chinese medicine (TCM) has achieved substantial results in the treatment of viral diseases (Pleschka et al., 2009; Ge et al., 2010). Modern studies have shown that TCM can not only directly inhibit viral replication but also regulate cellular and humoural immunity, improve pulmonary circulation, and eliminate and reduce respiratory inflammatory exudates (Ma et al., 2017; Shi et al., 2020), suggesting that TCM has the advantages and characteristics of multiple components, multiple targets, and integrated regulation. GHKGD is a clinical experience formula based on clinical characteristics and its performance in treating viral pneumonia in the Lingnan area (Zhao et al., 2018). This prescription is based on the initial formula Yin Qiao San for febrile disease, with the addition of *Ilex asprella* Champ. ex Benth., *Pogostemon cablin* (Blanco) Benth., *Notopterygium incisum* K.C.Ting ex H.T.Chang and other heat-reducing and damp-releasing drugs, including *Lonicera japonica* Thunb.*, Forsythia suspensa* (Thunb.) Vahl*, Saposhnikovia divaricata* (Turcz.) Schischk.*, Notopterygium incisum* K.C.Ting ex H.T.Chang.*, Bupleurum chinense* DC.*, Atractylodes lancea* (Thunb.) DC.*, Nepeta tenuifolia* Benth. *and Calculus bovis.* It has the functions of Shufeng Jiebiao, Qingre Jiedu, and Qushi Hezhong and is targeted for the treatment of wind, heat and wet invasion of lung-type influenza in Lingnan, with significant clinical effects (Yang et al., 2017a; Lyv et al., 2017).

In our previous studies, GHKGD could remarkly reduce the exudation of inflammatory mediators in mice with H1N1 (A/FM1/1/47) influenza virus pneumonia by improving anti-inflammatory cytokine levels (IL-10, IFN-γ), lowering pro-inflammatory cytokines (IL-6, TNF-α, MCP-1) (Chen et al., 2015). In addition, GHKGD could significantly prolong the average survival time of mice, and at the same time increase the oxygen content and blood oxygen saturation in the arterial blood of virus-infected mice, and reduce the partial pressure of carbon dioxide (Liu et al., 2015). However, the specific mechanism of GHKGD reducing the severity of pneumonia caused by influenza virus is not clear. Therefore, in this study, we try to use the strategy of network pharmacology, a recently developed discipline that combines holistic network analysis and pharmacology (Yue et al., 2017; Liu et al., 2020), to reveal its mechanism.

**FIGURE 1:**
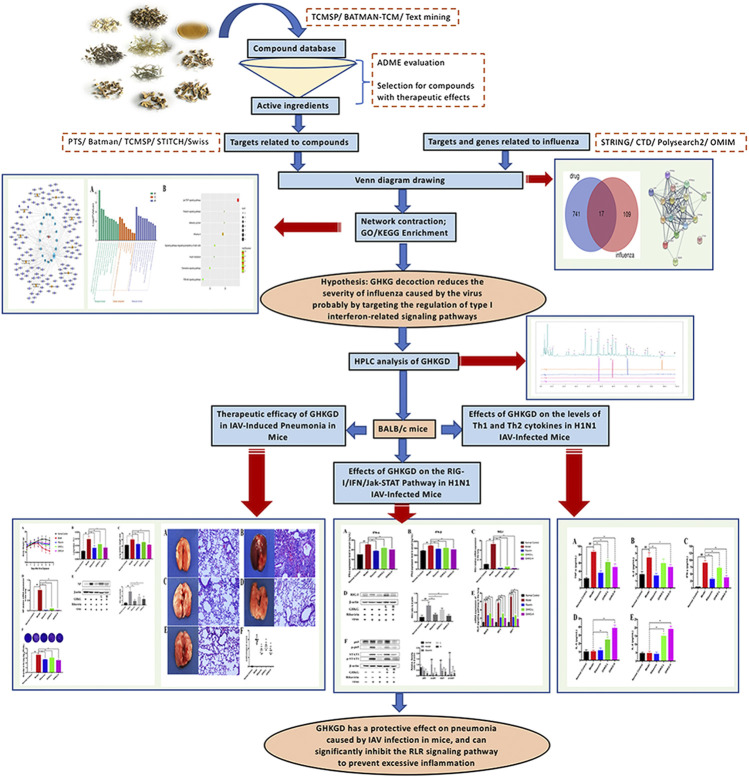
The technical strategy of the current study.

In this study, we used a network pharmacology method to analyse the interaction between active molecules, potential targets and target diseases of GHKGD. According to the preliminary analysis results, we studied the mechanism of GHKGD regulating the RLR signaling pathway in the treatment of influenza in a mouse model of IAV infection. The detailed technical strategy of the current study was shown in Figure 1.

## Materials and Methods

### Candidate Compound Screening

All of the chemical ingredients of GHKGD were collected from the Traditional Chinese Medicine Systems Pharmacology database and Analysis Platform (TCMSP, http://tcmspw.com) (Ru et al., 2014), Bioinformatics Analysis Tool for Molecular Mechanism of Traditional Chinese Medicine (BATMAN-TCM) (http://bionet.ncpsb.org/batman-tcm/index.php/Home/Index/index) (Liu et al., 2016) and wide-scale searches of the literature, including the Web of Science, PubMed, China BioMedical Literature (CBM) and China National Knowledge Infrastructure (CNKI) databases. Collectively, 714 compounds were identified. Here, we selected the ingredients that met the criteria of oral bioavailability (OB) ≥30% and drug-likeness (DL) ≥0.14 as candidate compounds for further analyses. OB is an important parameter to measure the pharmacokinetics and druggability of drugs *in vivo*. It represents the convergence of the processes of absorption, distribution, metabolism, and excretion (ADME) (Xu et al., 2012). DL is a qualitative principle used in drug design to accurately predict the “drug-like” nature of a compound (Lipinski et al., 2001).

### Identification of Drug Targets

To obtain as many drug compound targets as possible, we searched and predicted targets from multiple databases, including the Pharmaceutical Target Seeker (http://www.rcdd.org.cn/PTS/search) (Ding et al., 2017), TCMSP, BATMAN-TCM (http://bionet.ncpsb.org/batman-tcm/), Swiss Target Prediction (Swiss, http://www.swisstargetprediction.ch/) (Gfeller et al., 2014) and STITCH (http://stitch.embl.de/). All targets were restricted to human origin. Next, we retrieved the protein targets of influenza virus from several databases, such as the CTD database (http://ctdbase.org), OMIM database (https://omim.org/), Polysearch2 database (http://polysearch.cs.ualberta.ca/) and STRING database (https://string-db.org/cgi/input.pl). Then, all target names were converted into the corresponding official gene names in the UniProt database. The Venny2.1.0 online tool (http://bioinfogp.cnb.csic.es/tools/venny/index.html) was used to obtain the overlapping targets from the two sources to identify potential drug targets for the treatment of influenza.

### Gene Ontology Enrichment Analysis of Targets

To systematically understand the biological processes of GHKGD in the treatment of influenza, we performed gene ontology (GO) enrichment analysis of potential targets. The terms with a *p* value of less than 0.05 were selected for functional annotation and signaling pathway clustering. The above analysis was completed using the functional annotation tool of Metascape (https://metascape.org/gp/index.html#/main/step1).

### Protein-Protein Interaction Data

The Draw Venn Diagram tool (http://bioinformatics.psb.ugent.be/webtools/Venn/) was used to obtain the target genes for the interaction between the drug (Ganghuo Kanggan decoction) and the disease (influenza). The STRING search tool (version 11.0) (https://string-db.org/cgi/input.pl) was employed to show the interactions in the PPI data, with the species limited to “*Homo sapiens*.”

### Network Construction and Analysis

To comprehensively analyse the molecular mechanism of GHKGD in the treatment of influenza, two network diagrams, drug-target-disease and target-biological process diagrams, were constructed using Cytoscape 3.7.1 software. The NetworkAnalyzer tool in the software was used to analyse the network topology properties, and the Cytoscape plugin cytohubba was used to analyse the core nodes of the network.

### Preparation and HPLC Analysis of Ganghuo Kanggan Decoction

GHKGD is composed of 19 g *Ilex asprella* Champ. ex Benth., 6 g *Pogostemon cablin* (Blanco) Benth., 9 g *Forsythia suspensa* (Thunb.) Vahl, 6 g *Saposhnikovia divaricata* (Turcz.) Schischk., 5.5 g *Notopterygium incisum* K.C.Ting ex H.T.Chang, 9 g *Lonicera japonica* Thunb., 5 g *Bupleurum chinense* DC., 5 g *Atractylodes lancea* (Thunb.) DC., 5 g *Nepeta tenuifolia* Benth., 0.2 g artificial bezoar. All crude herbs were provided from the dispensary of the First Affiliated Hospital of Guangzhou University of Chinese Medicine. Firstly, all crude drugs except artificial bezoar powder were soaked in 1.5 L water for 2 h, then they were decocted to boiling for 1.5 h. The drugs were boiled once again for 1 h with 1 L water and the decoction was merged and filtered through a four-layer gauze. Next, the filtrates were concentrated to a concentration of 2.31 g crude drug/mL as the high dose and a concentration of 0.58 g crude drug/mL as the low dose. Finally, an artificial bezoar and volatile oil were added and mixed well. The decoction was stored at −4°C and sealed for later use *in vivo*. The positive drug ribavirin was purchased from Jiangxi Huiren Pharmaceutical Co., Ltd. (production batch number: 180401, specification: 100 mg/tablet).

HPLC-GHKGD analysis was performed at 246 nm for Prim-O-glucosylcimifugin, 5-O-methylvisammioside, Amygdalin, and Notopterol, using a Thermo Scientific U3000 HPLC system. Chromatographic separation was achieved with a Phenomenon® Luna-C18 analytical column (4.6 mm × 250 mm, 5 μm). Forsythin (lot numbers: 110821–201213, purity: 95.3%), Prim-O-glucosylcimifugin (lot numbers: 111522–201209, purity: 93.7%), 5-O-Methylvisammioside (lot numbers: 111523–201509, purity: 95.8%), Notoperol (lot numbers: 111820-201504, purity: 99.9%) were purchased from National Drug Reference Standards database. Took appropriate amounts of Prim-O-glucosylcimifugin, 5-O-Methylvisammioside, forsythin and Notoperol, and then added methanol to prepare solutions of 0.16, 0.20, 0.30, 0.20, 0.12 mg/mL, respectively. Took 10 mL of the GHKGD filtrate after decoction and filtration, evaporated it to dryness, added 1.5 g of neutral alumina, stirred evenly, passed through a column of neutral alumina column (100–200 mesh, 1.5 cm in diameter), and eluted with 100 mL of 70% ethanol. Collected the eluate, evaporated to dryness, and dissolved the residue with 50% methanol to 10 mL. Chromatographic condition: Phenomenon Luna C18 2) 100R (4.6 mm × 250 mm, 5 µm). Acetonitrile (A)- 0.2% glacial acetic acid (b) was taken as a mobile phase. The flow rate was 1.0 ml min^−1^, and the column temperature was 30°C. The detection wavelength was 246 nm. The injection volume was 10 µL.

### Animals and Experimental Groups

Sixty SPF BALB/c mice (30 males and 30 females) weighing between 16 g and 18 g were purchased from the Experimental Animal Center of Guangzhou University of Traditional Chinese Medicine (GZUCM). Animal experiments were approved by the Animal Care and Use Committee of the Experimental Animal Center of GZUCM. After 24 h quarantine, the mice were randomly divided into the normal control (NC) group, model (IAV-C + sterile saline, suspended in 0.5% Tween 80) group, positive control ribavirin group, GHKG high-dose group, and GHKG low-dose group, with 12 mice in each group. Standard mouse chow and tap water were provided ad libitum during the study. The mice were housed in the Guangdong Association for the Accreditation of Laboratory Animal Care-accredited GZUCM Laboratory Animal Research Center.

All mice except those in the normal control group were infected with two LD_50_ H1N1 (PR8) virus by intranasal instillation under mild anaesthesia using ether. Mice in the normal control group were administered an equal volume of 0.9% sodium chloride solution by intranasal instillation. Two hours after infection, drugs were administered orally to the treatment groups. The ribavirin was administered at 75 mg/kg/day, and the GHKGD was administered to the high-dose and low-dose groups at 46.2 g/kg/day and 11.6 g/kg/day, respectively. The normal control and model groups were given an equal volume of 0.9% sodium chloride solution. Dosing was continued once daily for 5 days. The disease status of the mice was observed daily, and the body weight was recorded. On day 5 after infection, mice were sacrificed to collect relevant samples, and the body weight, wet lung weight, and extent of lung pathological changes were measured.

### Viruses and Cells

Influenza A/Puerto Rico/8/34 (H1N1) virus was maintained at the Laboratory Animal Center, Guangzhou University of Chinese Medicine, and propagated in embryonated chicken eggs. The viruses were aliquoted and stored at −80°C. Madin-Darby canine kidney (MDCK) cells were maintained in minimum essential medium (MEM) containing 10% foetal bovine serum and antibiotics (penicillin and streptomycin).

### Plague Reduction Assay

MDCK cells were seeded in 6-well tissue culture plates (6 × 10^5^ cells/well) and were then incubated at 37°C in 5% CO_2_ for 24 h. The cells were washed once with PBS and were then infected with the collected mouse lung tissue supernatant and incubated at 37°C for 2 h. After washing three times with PBS, the cell monolayers were covered with agar overlay medium (MEM supplemented with 1% low melting point agarose and 2.5 μg/mL TPCK-treated trypsin) and incubated at 37°C for 3–4 days. The cell monolayers were fixed with 4% paraformaldehyde for 1 h. The covering was then removed, and the cell monolayers were stained with 2% crystal violet solution containing 10% ethanol.

### Histopathological Staining

Mouse lung tissue was stored in a 5 mL EP tube filled with 4% paraformaldehyde solution, and after fixation for 48 h, sections were stained with haematoxylin and eosin (HE) and then tested under a microscopy in a double-blinded manner. The following scoring criteria were used to classify the degree of lung injury into five levels: the presence of necrotic bronchiole and bronchial epithelium; exudate of plasma cells in the bronchiole and bronchial lumen; inflammatory cells in the bronchiolar, peribronchiolar and alveolar interstitium (predominantly lymphocytes and neutrophils); collapse of the alveoli or bronchi (atelectasis); and diffuse or multifocal interstitial edema. No damage is marked as 0, mild damage is 1, moderate damage is 2, severe damage is 3, and severe histological change is 4 (Parsey et al., 1998).

### ELISA

The IFN-α and IFN-β levels in mouse serum were measured using ELISA according to the manufacturer’s instructions. Each treatment was analysed in triplicate. A Mouse IFN-α SimpleStep ELISA^®^ Kit (Cat. Number: ab252352) and a Mouse IFN-β SimpleStep ELISA® Kit (Cat. Number: ab252363) were purchased from Abcam (Burlingame, CA, USA).

### Real-Time QPCR Analysis

The mRNA expression levels of the NP, RIG-I, MAVS, IRF3, IRF7 and STAT1 genes in lung homogenates were detected by real-time quantitative PCR. Total RNA from mouse lungs was extracted using an Ultrapure RNA kit (CoWin Biotech, Beijing, China), and cDNA was then synthesized from the total RNA using a M-MLV Reverse transcriptase kit (Promega, Madison, WI, United States).

### Western Blot Analysis

Western blot analysis was performed according to standard procedures. In brief, tissues were lysed in radio-immunoprecipitation assay (RIPA) buffer containing 1% PMSF and were then centrifuged at 10,000 rpm for 10 min to remove insoluble matter. The protein concentration was determined using a BCA protein concentration assay kit. Equal amounts of protein (30 μg) were separated via 10% SDS-PAGE and transferred to a PVDF membrane (Mannheim, Germany). The membrane was incubated with 5% skim milk to block non-specific binding sites, incubated with the primary antibody overnight at 4°C, and then incubated with the corresponding horseradish peroxidase (HRP)-conjugated secondary antibody at room temperature for 1–2 h. ECL reagent (Rockford, IL, USA) was used to detect antigen-antibody complexes. The protein expression levels were normalized to that of β-actin in the same sample.

### BD Cytometric Bead Arry Analysis

The levels (pg/ml) of tumor necrosis factor-α (TNF-α), interferon-γ (IFN-γ), interleukin-2 (IL-2), interleukin-4 (IL-4) and interleukin-5 (IL-5) in serum samples were determined using a BD™ Cytometric Bead Array Mouse Th1/Th2 Cytokine Kit (CBA) (lot: 9073921, BD Biosciences Pharmingen, San Diego, USA), according to the manufacturer’s instructions. Fluorescence was determined using a flow cytometer (FACS Calibur, Becton-Dickinson Bio-sciences, Heidelberg, Germany) and cytokine level was analyzed using a BD CBA Software.

### Statistical Analysis

All data in this experiment are expressed as the mean ± SEM values. Multiple statistical analyses were conducted by one-way analysis of variance (ANOVA). Comparisons between two groups were performed using Dunnett's *t*-test. A probability value of *p* < 0.05 was defined as significant. GraphPad Prism 7.0 was used for statistical analyses.

## Results

### Compound Information

The compounds comprising the 11 kinds of Chinese medicine in Gangzhi Kanggan Decoction were obtained from the TCMSP database and the BATMAN-TCM database. A total of 182 candidate compounds were obtained after screening with ADME parameters (OB ≥ 30%, DL ≥ 0.14). Certain compounds with significant pharmacological effects and high levels were also considered. The final candidate compounds are listed in Supplementary Table S1.

### Compouds in GHKGD Active Against Influenza

Based on the CTD, Polysearch2, OMIM and STRING databases, a total of 126 (Supplementary Table S2) targets directly and indirectly associated with influenza were obtained. By employing three available resources, namely, the PTS, BATMAN, and TCMSP databases, we obtained 798 GHKGD-related targets (Supplementary Table S3). After determination of the overlapping targets obtained from these two sources, a total of 17 potential target gene-associated compounds were obtained. Furthermore, 116 compounds in GHKT decoction active against influenza were retrieved. Detailed information on the potential protein targets and active compounds in GHKGD obtained are presented in Tables 1,2, respectively.

**TABLE 1:** Information on 17 potential target gene-associated compounds.

Uniprot ID	Protein name	Gene name	Degree
P00533	Epidermal growth factor receptor	EGFR	132
P52333	Tyrosine-protein kinase JAK3	JAK3	39
P18031	Tyrosine-protein phosphatase non-receptor type 1	PTPN1	16
O60674	Tyrosine-protein kinase JAK2	JAK2	11
Q96AZ6	Interferon-stimulated gene 20 kDa protein	ISG20	11
P17706	Tyrosine-protein phosphatase non-receptor type 2	PTPN2	8
P40763	Signal transducer and activator of transcription 3	STAT3	7
Q06124	Tyrosine-protein phosphatase non-receptor type 11	PTPN11	7
P42224	Signal transducer and activator of transcription 1-alpha/beta	STAT1	7
P23458	Tyrosine-protein kinase JAK1	JAK1	7
P29597	Non-receptor tyrosine-protein kinase TYK2	TYK2	6
P19525	Interferon-induced, double-stranded RNA-activated protein kinase	EIF2AK2	6
P10619	Lysosomal protective protein	CTSA	5
P40238	Thrombopoietin receptor	MPL	4
Q14164	Inhibitor of nuclear factor kappa-B kinase subunit epsilon	IKBKE	4
P05412	Transcription factor AP-1	JUN	3
P29350	Tyrosine-protein phosphatase non-receptor type 6	PTPN6	2

**TABLE 2:** 116 active compounds in GHKG decoction related to influenza.

Mol ID	Molecule name	MW	OB (%)	DL	Herb	Degree
MOL008838	Methyl (4R)-4-[(3R,5S,7S,8R,9S,10S,12S,13R,14S,17R)-3,7,12-trihydroxy-10,13-dimethyl-2,3,4,5,6,7,8,9,11,12,14,15,16,17-tetradecahydro-1H-cyclopenta[a]phenanthren-17-yl]pentanoate	422.67	32.32	0.76	*Calculus bovis*	3
MOL008839	Methyl desoxycholate	406.67	34.63	0.73	*Calculus bovis*	3
MOL008845	Deoxycholic acid	392.64	40.72	0.68	*Calculus bovis*	2
MOL008846	ZINC01280365	330.51	46.38	0.49	*Calculus bovis*	2
MOL000953	CLR	386.73	37.87	0.68	*Calculus bovis*	2
MOL001645	Linoleyl acetate	308.56	42.1	0.2	*Bupleurum chinense* DC.	3
MOL001789	Isoliquiritigenin	256.27	85.32	0.15	*Bupleurum chinense* DC.;	9
MOL002776	Baicalin	446.39	40.12	0.75	*Bupleurum chinense* DC.	3
					*Lonicera japonica* thunb.;	
					*Bupleurum chinense* DC.;	
MOL000449	Stigmasterol	412.77	43.83	0.76	*Nepeta tenuifolia* benth.	4
MOL000354	Isorhamnetin	316.28	49.6	0.31	*Bupleurum chinense* DC.	2
					*Lonicera japonica* thunb.;	
					*Forsythia suspensa* (thunb.) vahl;	
MOL000422	Kaempferol	286.25	41.88	0.24	*Bupleurum chinense* DC.	4
MOL004609	Areapillin	360.34	48.96	0.41	*Bupleurum chinense* DC.	2
MOL013187	Cubebin	356.4	57.13	0.64	*Bupleurum chinense* DC.	3
MOL004624	Longikaurin	348.48	47.72	0.53	*Bupleurum chinense* DC.	2
MOL004628	Octalupine	264.41	47.82	0.28	*Bupleurum chinense* DC.	2
MOL004644	Sainfuran	286.3	79.91	0.23	*Bupleurum chinense* DC.	2
MOL004648	Troxerutin	346.56	31.6	0.28	*Bupleurum chinense* DC.	3
MOL004653	(+)-anomalin	426.5	46.06	0.66	*Bupleurum chinense* DC.	3
MOL004683	Methyl (2E,4E)-octadeca-2,4-dienoate	294.53	38.77	0.17	*Bupleurum chinense* DC.	2
MOL004702	Saikosaponin c_qt	472.78	30.5	0.63	*Bupleurum chinense* DC.	3
MOL004718	α-spinasterol	412.77	42.98	0.76	*Bupleurum chinense* DC.	2
					*Bupleurum chinense* DC.;	
					*Pogostemon cablin* (blanco) benth.;	
					*Lonicera japonica* thunb.;	
					*Forsythia suspensa* (thunb.) vahl;	
MOL000098	Quercetin	302.25	46.43	0.28	*Nepeta tenuifolia* benth.	6
MOL000181	Atractylenolide III	248.35	31.15	0.17	*Atractylodes lancea* (thunb.) DC.	2
MOL000085	Beta-daucosterol_qt	414.79	36.91	0.75	*Atractylodes lancea* (thunb.) DC.	3
MOL000088	Beta-sitosterol 3-O-glucoside_qt	414.79	36.91	0.75	*Atractylodes lancea* (thunb.) DC.	2
MOL000092	daucosterin_qt	414.79	36.91	0.76	*Atractylodes lancea* (thunb.) DC.	2
MOL000094	daucosterol_qt	414.79	36.91	0.76	*Atractylodes lancea* (thunb.) DC.	2
MOL000043	Atractylenolide i	230.33	37.37	0.15	*Atractylodes lancea* (thunb.) DC.	5
MOL000184	NSC63551	412.77	39.25	0.76	*Atractylodes lancea* (thunb.) DC.	2
MOL000188	3β-acetoxyatractylone	274.39	40.57	0.22	*Atractylodes lancea* (thunb.) DC.	2
MOL000186	Stigmasterol	412.77	43.83	0.76	*Atractylodes lancea* (thunb.) DC.	2
MOL000179	2-Hydroxyisoxypropyl-3-hydroxy-7-isopentene-2,3-dihydrobenzofuran-5-carboxylic	306.39	45.2	0.2	*Atractylodes lancea* (thunb.) DC.	4
MOL000180	Aractylenolide II	232.35	46.2	0.15	*Atractylodes lancea* (thunb.) DC.	4
MOL000044	atractylenolideII	232.35	47.5	0.15	*Atractylodes lancea* (thunb.) DC.	3
MOL000011	AIDS-227003	386.38	68.83	0.66	*Saposhnikovia divaricata* (turcz.) schischk.	2
MOL011730	11-hydroxy-sec-o-beta-d-glucosylhamaudol_qt	292.31	50.24	0.27	*Saposhnikovia divaricata* (turcz.) schischk.	2
MOL011732	Anomalin	426.5	59.65	0.66	*Saposhnikovia divaricata* (turcz.) schischk.	2
MOL011737	Divaricatacid	320.32	87	0.32	*Saposhnikovia divaricata* (turcz.) schischk.	2
MOL011740	Divaricatol	334.35	31.65	0.38	*Saposhnikovia divaricata* (turcz.) schischk.	2
					*Saposhnikovia divaricata* (turcz.) schischk.;	
MOL001941	Ammidin	270.3	34.55	0.22	*Notopterygium incisum* K.C.Ting ex H.T.Chang.	3
MOL011746	Isopimpinellin	246.23	43.14	0.17	*Saposhnikovia divaricata* (turcz.) schischk.	9
MOL011747	Ledebouriellol	374.42	32.05	0.51	*Saposhnikovia divaricata* (turcz.) schischk.	2
MOL011749	Phelloptorin	300.33	43.39	0.28	*Saposhnikovia divaricata* (turcz.) schischk.	2
MOL011754	4-hydroxy-9-methoxyfuro[3,2-g]chromen-7-one	232.2	31.78	0.15	*Saposhnikovia divaricata* (turcz.) schischk.	9
MOL011755	5-methoxy-8-hydroxypsoralen	232.2	48.4	0.15	*Saposhnikovia divaricata* (turcz.) schischk.	8
MOL001944	Marmesin	246.28	50.28	0.18	*Saposhnikovia divaricata* (turcz.) schischk.;	8
					*Notopterygium incisum* K.C.Ting ex H.T.Chang.	
MOL002644	Phellopterin	300.33	40.19	0.28	*Saposhnikovia divaricata* (turcz.) schischk.;	4
					*Notopterygium incisum* K.C.Ting ex H.T.Chang.	
					*Saposhnikovia divaricata* (turcz.) schischk.;	
MOL000359					*Nepeta tenuifolia* benth.;	
	Sitosterol	414.79	36.91	0.75	*Notopterygium incisum* K.C.Ting ex H.T.Chang.	4
					*Saposhnikovia divaricata* (turcz.) schischk.;	
					*Lonicera japonica* thunb.;	
					*Forsythia suspensa* (thunb.) vahl;	
MOL000358	Beta-sitosterol	414.79	36.91	0.75	*Notopterygium incisum* K.C.Ting ex H.T.Chang.	6
MOL001494	Mandenol	308.56	42	0.19	*Saposhnikovia divaricata* (turcz.) schischk.;	4
					*Lonicera japonica* thunb.	
MOL001889	Methyl linolelaidate	294.53	41.93	0.17	*Saposhnikovia divaricata* (turcz.) schischk.	2
					*Saposhnikovia divaricata* (turcz.) schischk.;	
MOL001942	Isoimperatorin	270.3	45.46	0.23	*Notopterygium incisum* K.C.Ting ex H.T.Chang.	4
MOL003588	Prangenidin	270.3	36.31	0.22	*Saposhnikovia divaricata* (turcz.) schischk.	2
					*Saposhnikovia divaricata* (turcz.) schischk.;	
MOL004793	Marmesine	246.28	84.77	0.18	*Notopterygium incisum* K.C.Ting ex H.T.Chang.	3
MOL007514	Methyl icosa-11,14-dienoate	322.59	39.67	0.23	*Saposhnikovia divaricata* (turcz.) schischk.	3
MOL011648	METHYL 10-OCTADECENOATE	296.55	31.9	0.17	*Saposhnikovia divaricata* (turcz.) schischk.	2
MOL013077	Decursin	328.39	39.27	0.38	*Saposhnikovia divaricata* (turcz.) schischk.	2
MOL002707	Phytofluene	543.02	43.18	0.5	*Lonicera japonica* thunb.	3
MOL002773	Beta-carotene	536.96	37.18	0.58	*Lonicera japonica* thunb.	3
MOL000432	Linolenic acid	278.48	45.01	0.15	*Nepeta tenuifolia* benth.	
					*Lonicera japonica* thunb.;	
					*Forsythia suspensa* (thunb.) vahl;	
MOL000006	Luteolin	286.25	36.16	0.25	*Nepeta tenuifolia* benth.	4
MOL002523	()-Cyclosativene	204.39	33.43	0.15	*Pogostemon cablin* (blanco) benth.	2
MOL002879	Diop	390.62	43.59	0.39	*Pogostemon cablin* (blanco) benth.	2
MOL005884	Patchoulan 1,12-diol	262.43	38.17	0.25	*Pogostemon cablin* (blanco) benth.	2
MOL005890	Pachypodol	356.4	75.06	0.4	*Pogostemon cablin* (blanco) benth.	2
MOL005907	ZINC02090576	220.39	77.74	0.16	*Pogostemon cablin* (blanco) benth.	2
MOL005921	Quercetin 7-O-β-D-glucoside	300.28	49.57	0.27	*Pogostemon cablin* (blanco) benth.	2
MOL005922	Acanthoside B	580.64	43.35	0.77	*Pogostemon cablin* (blanco) benth.	3
MOL005923	3,23-dihydroxy-12-oleanen-28-oic acid	518.56	30.86	0.86	*Pogostemon cablin* (blanco) benth.	2
MOL000695	Patchouli alcohol	222.41	101.96	0.14	*Pogostemon cablin* (blanco) benth.	2
MOL003062	, 5′-retro-beta,beta-carotene-3,3′-dione, 4′,5′-didehydro-	562.9	31.22	0.55	*Lonicera japonica* thunb.	3
MOL003044	Chryseriol	300.28	35.85	0.27	*Lonicera japonica* thunb.	2
MOL002914	Eriodyctiol (flavanone)	288.27	41.35	0.24	*Lonicera japonica* thunb.	2
MOL003103	Methyl octadeca-8,11-dienoate	294.53	41.93	0.17	*Lonicera japonica* thunb.	2
MOL003036	ZINC03978781	412.77	43.83	0.76	*Lonicera japonica* thunb.	2
MOL001495	Ethyl linolenate	306.54	46.1	0.2	*Lonicera japonica* thunb.	3
MOL003101	7-epi-vogeloside	432.47	46.13	0.58	*Lonicera japonica* thunb.	3
MOL003059	Kryptoxanthin	552.96	47.25	0.57	*Lonicera japonica* thunb.	2
MOL003014	Secologanic dibutylacetal_qt	384.57	53.65	0.29	*Lonicera japonica* thunb.	3
MOL003006	(-)-(3R,8S,9R,9aS,10aS)-9-ethenyl-8-(beta-D-glucopyranosyloxy)-2,3,9,9a,10,10a-hexahydro-5-oxo-5H,8H-pyrano[4,3-d]oxazolo[3,2-a]pyridine-3-carboxylic acid_qt	281.29	87.47	0.23	*Lonicera japonica* thunb.	2
MOL003315	3beta-acetyl-20,25-epoxydammarane-24alpha-ol	502.86	33.07	0.79	*Forsythia suspensa* (thunb.) vahl	2
MOL000522	Arctiin	534.61	34.45	0.84	*Forsythia suspensa* (thunb.) vahl	3
MOL003305	PHILLYRIN	534.61	36.4	0.86	*Forsythia suspensa* (thunb.) vahl	2
MOL003281	20 (S)-dammar-24-ene-3β,20-diol-3-acetate	486.86	40.23	0.82	*Forsythia suspensa* (thunb.) vahl	2
MOL003365	Lactucasterol	426.75	40.99	0.85	*Forsythia suspensa* (thunb.) vahl	2
MOL003344	β-amyrin acetate	468.84	42.06	0.74	*Forsythia suspensa* (thunb.) vahl	2
MOL003347	hyperforin	536.87	44.03	0.6	*Forsythia suspensa* (thunb.) vahl	2
MOL003348	Adhyperforin	550.9	44.03	0.61	*Forsythia suspensa* (thunb.) vahl	2
MOL003290	(3R,4R)-3,4-bis[(3,4-dimethoxyphenyl)methyl]oxolan-2-one	386.48	52.3	0.48	*Forsythia suspensa* (thunb.) vahl	2
MOL003295	(+)-pinoresinol monomethyl ether	372.45	53.08	0.57	*Forsythia suspensa* (thunb.) vahl	2
MOL000211	Mairin	456.78	55.38	0.78	*Forsythia suspensa* (thunb.) vahl	2
MOL003308	(+)-pinoresinol monomethyl ether-4-D-beta-glucoside_qt	372.45	61.2	0.57	*Forsythia suspensa* (thunb.) vahl	2
MOL003283	(2R,3R,4S)-4-(4-hydroxy-3-methoxy-phenyl)-7-methoxy-2,3-dimethylol-tetralin-6-ol	360.44	66.51	0.39	*Forsythia suspensa* (thunb.) vahl	2
MOL003370	Onjixanthone I	302.3	79.16	0.3	*Forsythia suspensa* (thunb.) vahl	2
MOL003322	FORSYTHINOL	372.45	81.25	0.57	*Forsythia suspensa* (thunb.) vahl	2
MOL003306	ACon1_001697	372.45	85.12	0.57	*Forsythia suspensa* (thunb.) vahl	2
MOL003358	Euxanthone	228.21	92.98	0.16	*Forsythia suspensa* (thunb.) vahl	14
MOL003330	(−)-Phillygenin	372.45	95.04	0.57	*Forsythia suspensa* (thunb.) vahl	2
					*Notopterygium incisum* K.C.Ting ex H.T.Chang.;	
MOL001941	Ammidin	270.3	34.55	0.22	*Saposhnikovia divaricata* (turcz.) schischk.	3
MOL011962	6′-feruloylnodakenin	584.62	32.02	0.67	*Notopterygium incisum* K.C.Ting ex H.T.Chang.	3
MOL011963	8-geranoxy-5-methoxypsoralen	368.46	40.97	0.5	*Notopterygium incisum* K.C.Ting ex H.T.Chang.	3
MOL011968	Coumarin, glycoside	534.61	33.07	0.78	*Notopterygium incisum* K.C.Ting ex H.T.Chang.	4
MOL011969	Demethylfuropinnarin	270.3	41.31	0.21	*Notopterygium incisum* K.C.Ting ex H.T.Chang.	2
MOL011971	diversoside_qt	332.43	67.57	0.31	*Notopterygium incisum* K.C.Ting ex H.T.Chang.	2
MOL011975	Notoptol	354.43	62.97	0.48	*Notopterygium incisum* K.C.Ting ex H.T.Chang.	3
MOL011976	2-(4-hydroxyphenyl)ethyl 4-methoxybenzoate	272.32	36.63	0.17	*Notopterygium incisum* K.C.Ting ex H.T.Chang.	7
MOL011980	Pterostilbene	256.32	77.54	0.14	*Notopterygium incisum* K.C.Ting ex H.T.Chang.	13
MOL001951	Bergaptin	338.43	41.73	0.42	*Notopterygium incisum* K.C.Ting ex H.T.Chang.	3
MOL001956	Cnidilin	300.33	32.69	0.28	*Notopterygium incisum* K.C.Ting ex H.T.Chang.	2
MOL003609	(8S)-8-(2-hydroxypropan-2-yl)-8,9-dihydrofuro[2,3-h]chromen-2-one	246.28	32.11	0.17	*Notopterygium incisum* K.C.Ting ex H.T.Chang.	2
MOL004792	Nodakenin	408.44	57.12	0.69	*Notopterygium incisum* K.C.Ting ex H.T.Chang.	2
MOL002881	Diosmetin	300.28	31.14	0.27	*Nepeta tenuifolia* benth.	
MOL011849	Schizonepetoside B	330.42	31.02	0.28	*Nepeta tenuifolia* benth.	2
MOL011856	Schkuhrin I	420.5	54.45	0.52	*Nepeta tenuifolia* benth.	3
MOL005100	5,7-dihydroxy-2-(3-hydroxy-4-methoxyphenyl)chroman-4-one	302.3	47.74	0.27	*Nepeta tenuifolia* benth.	
MOL001506	Supraene	410.8	33.55	0.42	*Nepeta tenuifolia* benth.	3
MOL005043	Campest-5-en-3beta-ol	400.76	37.58	0.71	*Nepeta tenuifolia* benth.	2
MOL1	19-Dehydroursolic acid	—	—	—	*Ilex asprella* champ. ex benth.	3
MOL2	Oblonganoside B	—	—	—	*Ilex asprella* champ. ex benth.	3
MOL3	Ilexolic acid	—	—	—	*Ilex asprella* champ. ex benth.	3
MOL000263	Oleanic acid	456.78	29.02	0.76	*Ilex asprella* champ. ex benth.	3
MOL000414	Caffeic acid	180.17	54.97	0.05	*Ilex asprella* champ. ex benth.	3
MOL000511	Ursolic acid	456.78	16.77	0.75	*Ilex asprella* champ. ex benth.	3
MOL004550	Pomolic acid	472.78	16.85	0.73	*Ilex asprella* champ. ex benth.	3

### Protein-Protein Interaction Network of Target Genes

As shown in Figure 1A, there were 17 active components of GHKGD acting on influenza-related targets: MPL, EIF2AK2, PTPN1, PTPN11, EGFR, TYK2, ISG20, IKBKE, PTPN6, PTPN2, JAK3, JAK1, JAK2, STAT3, CTSA, JUN, and STAT1. The PPI relationships among these 17 target genes were obtained by the STRING tool (Figure 1B). The PPI relationship network had 17 nodes and 77 edges, and the average node degree was 9.06.

### Gene Ontology Enrichment Analysis for Potential Targets in Influenza

**FIGURE 2:**
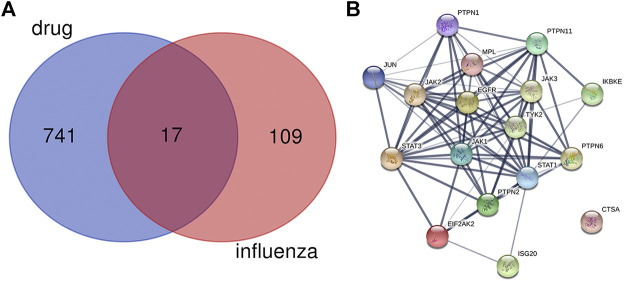
Protein-protein interaction (PPI) networks of ingredients of GHKGD with activity against influenza. **(A)** The intersection of the drug (GHKGD) targets and influenza-related targets. There were 17 relevant overlapping targets, and all are shown on the right. **(B)** Each node represents the relevant gene, and the line thickness of the edges indicates the strength of data support.

To explore the molecular mechanism of GHKGD in influenza, GO enrichment analysis and KEGG enrichment were performed on the 17 candidate targets. A total of 81 biological processes, 10 cellular components, and 30 molecular functions were obtained by GO analysis, from which the top 10 terms were selected (*p* < 0.05) (Figure 2A). In addition, eight pathways were obtained by KEGG analysis, which were ralated to influenza (*p* < 0.05) (Figure 2B). These biological processes might be important for the occurrence and development of influenza and included terms such as regulation of type I interferon-mediated signaling pathway, regulation of interferon-gamma-mediated signaling pathway, negative regulation of cell proliferation, type I interferon signaling pathway, Jak-STAT signaling pathway, Chemokine signaling pathway, and PI3K-Akt signaling pathway. These preliminary results support the hypothesis that GHKGD reduces the severity of influenza caused by the virus, at least in part through targeting the regulation of type I interferon-related signaling pathways and the Jak-STAT signaling pathway.

### Interaction Network Contruction and Network Analysis

**FIGURE 3:**
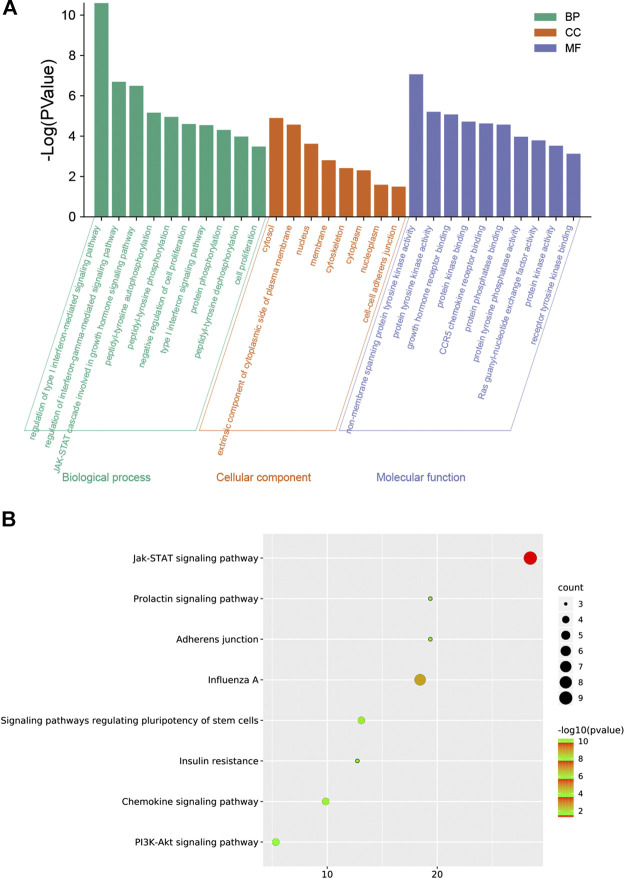
GO enrichment analysis by Metascape database. **(A)** Heatmap of GO enrichment. **(B)** GO-genes chord.

To reveal the synergistic multi-component and multi-target effects of GHKGD in the treatment of influenza as well as to explore its mechanism of action, a compound-target-disease (C-T-D) network was constructed and analysed. This network was composed of 151 nodes (116 compounds and 17 target genes) and 22,650 edges (Figure 3). The network degree of heterogeneity was 1.862, and the network centrality was 0.682. Analysing the topological parameters of the network helps identify core nodes, which are compounds and targets that play an important role in the network. Here, we used the node degree to identify important components and kernel targets. The size of a node is directly proportional to its degree. The larger the node, the higher is the degree, and the more important it is in the network. As shown in Figure 1, the top 10 target genes ranked by node degree were EGFR (106), JAK3 (35), PTPN1 (16), JAK2 (9), ISG20 (9), PTPN2 (8), STAT3 (7), PTPN11 (7), STAT1 (7), and JAK1 (7). In addition, the core compounds (degree ≥ 8) included MOL003358 (14), MOL011980 (13), MOL017746 (9), MOL11754 (9), MOL001789 (9), MOL011755 (8), MOL000358 (8) and MOL001944 (8). Some compounds, such as compound MOL003358, can act on multiple targets simultaneously, while some targets, such as EGFR, JAK3, and ISG20, can be acted on by multiple compounds simultaneously. This pattern explains the multi-component and multi-target characteristics of TCMs. Some compounds, such as compounds MOL001789 (isoliquiritigenin), MOL000098 (quercetin), MOL000006 (luteolin) and MOL000422 (kaempferol), have been reported to exhibit activity against the influenza virus (Traboulsi et al., 2015; Zhang et al., 2017; Yan et al., 2019).

### HPLC Analysis of Ganghuo Kanggan Decoction

**FIGURE 4:**
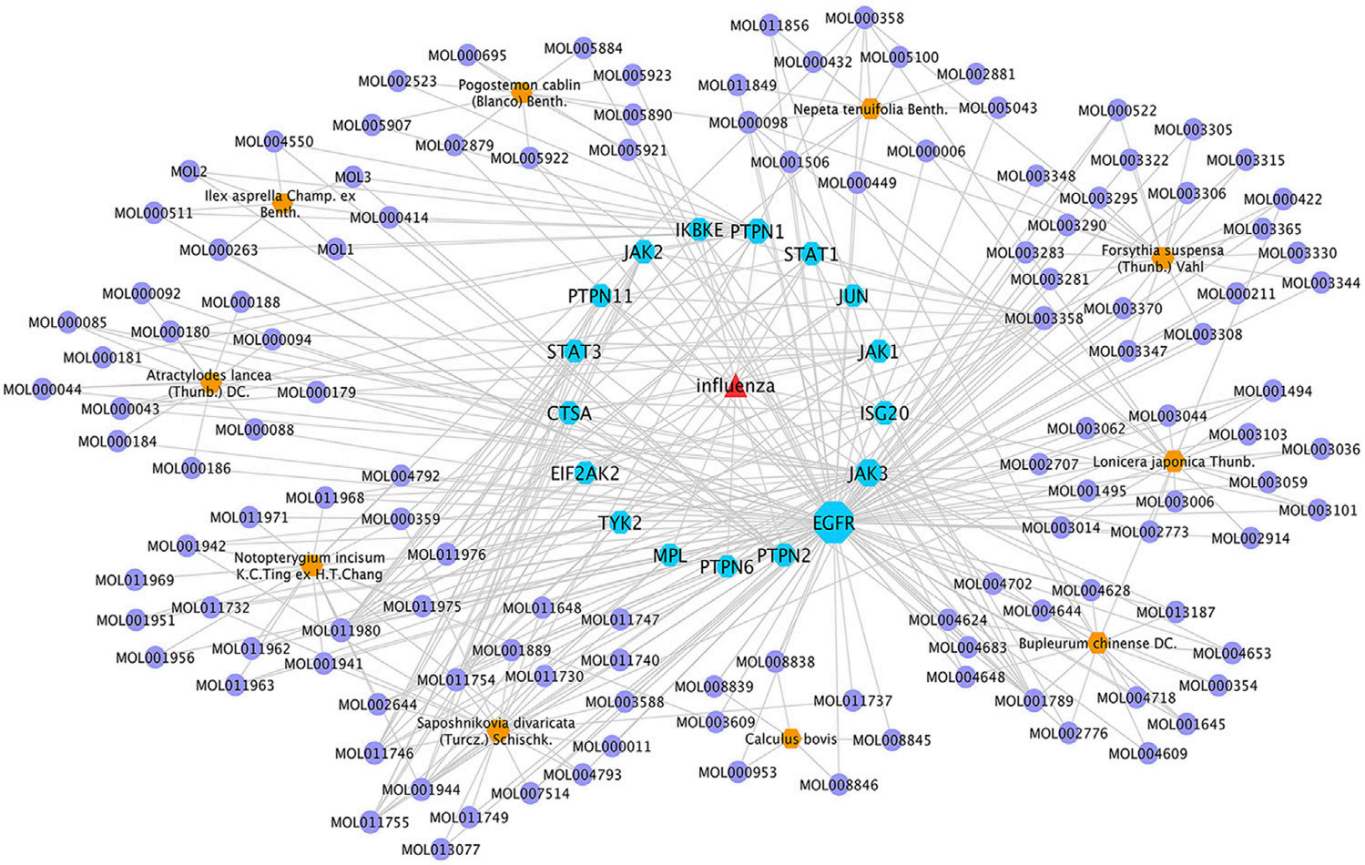
GHKGD component-target-disease network. The network was constructed to reveal interactions between active compounds and potential targets of GHKGD. Red triangular nodes represent the disease, blue octagonal nodes represent genes, yellow hexagonal nodes represent Chinese medicines, and purple circular nodes represent active ingredients. The size of each target is positively related to its degree in the network.

The results of representative HPLC analysis were shown in Figure 4.

### Therapeutic Efficacy of Ganghuo Kanggan Decoction in IAV-Induced Pneumonia in Mice

**FIGURE 5:**
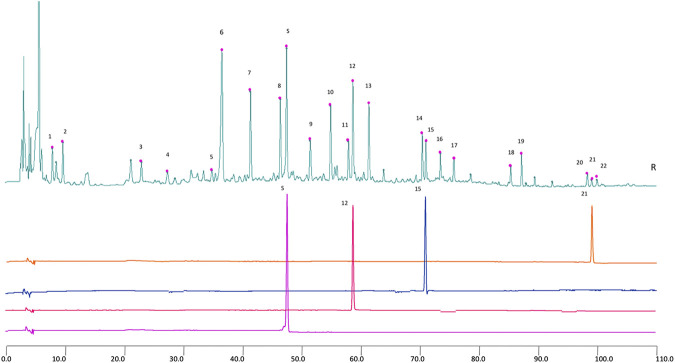
2D HPLC chromatograms of MHT water extract used in the trial. X-axis is the retention time, and Y-axis is the absorbance unit. S. Prim-O-glucosylcimifugin, 12. 5-O-methylvisammioside, 15. Amygdalin, and 21. Notopterol.

We evaluated the therapeutic effect of the drug in murine pneumonia caused by IAV at doses not inducing significant clinical symptoms and weight changes (high dose, 46.2 g/kg; low dose, 11.6 g/kg). As shown in Figure 5A, administration of GHKGD effectively protected the infected mice from weight loss caused by IAV infection. Although initial weight loss occurred in each group beginning on day 3 of infection, the weight reduction trend over the next 3 days observed for mice treated with GHKG was similar to that observed for mice in the ribavirin group, while untreated mice (model group) showed significant weight loss over the next 3 days. These results indicated that treatment with GHKG effectively protected mice against weight loss caused by IAV infection.

The lung index and lung wet/dry weight ratio were calculated to assess the severity of influenza viral pneumonia. The pulmonary index is an indicator of the severity of pneumonia, and it increases significantly in the early stages of IAV infection (Barnard, 2009; Dai et al., 2014). As shown in Figures 5B,C, the lung index of the model group was significantly higher than that of the normal control group (*p* < 0.01). The lung index of the model group was 0.1466 ± 0.0022. In the groups treated with 46.2 and 11.6 g/kg GHKGD, the lung indices were 0.00840 ± 0.0012 and 0.00842 ± 0.0007, respectively, and were similar to that of the ribavirin group (0.00824 ± 0.0019). The lung wet/dry weight ratio showed a similar change trend. Both the high and low doses of GHKGD significantly reduced the lung wet/dry weight ratio of H1N1-infected mice (*p* < 0.01).

To further study the efficacy of GHKGD against H1N1, we measured the mRNA and protein levels of NP genes and their viral titers in mouse lung tissues on the fifth day after infection (Figures 5D–F). The NP mRNA expression level in the lung tissue of the model group (1 ± 0.11373) was 5555 times higher than that of the normal control group (0.00018 ± 0.00002), indicating that the model was successfully established. Both ribavirin and GHKGD showed excellent effects on reducing viral NP mRNA expression. It is worth noting that the therapeutic effect of high-dose GHKGD (0.01972 ± 0.001470) was similar to that of ribavirin (0.02401 ± 0.00222) and was 50 times lower than that observed in the model group. The change in NP protein expression was consistent with the change in mRNA expression. The virus titer reflects the threshold level of virus required to cause infection and the ability to resist the virus (Ma et al., 2018). The virus titer in lung tissue in the model group was 3.66 ± 0.31 log_10_ PFU/mL. Compared with that in the model group, the virus titers in the lungs of virus-infected mice treated with GHKGD (46.2 or 11.6 g/kg/day) and ribavirin were significantly reduced (*p* < 0.01) (Figure 5F). This result was consistent with the results of lung histological changes.

**FIGURE 6:**
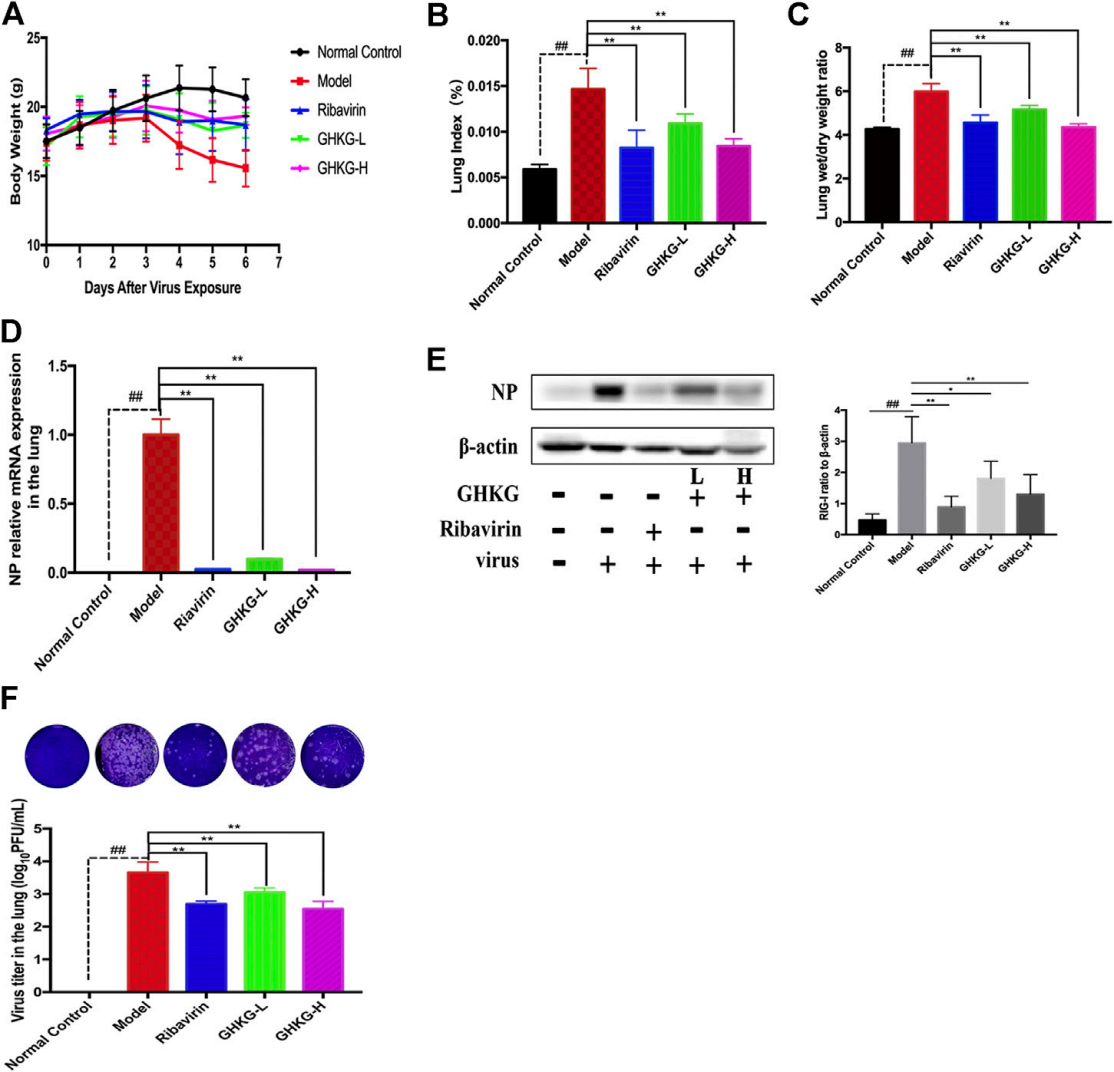
Evaluation of the therapeutic effect of GHKGD in H1N1‐infected mice. **(A)** Body weight change (mean ± SD). Mice were infected with influenza A/Puerto Rico/8/34 (H1N1) virus (2 LD 50 ) and treated with GHKGD high (46.2 g/kg/d) or low (11.6 g/kg/d) dose or ribavirin (75 mg/kg/d) once a day for 5 days. Clinical signs were observed for 6 days (n = 6). Mice were sacrificed at 5 dpi. The lungs were removed and rinsed with sterile PBS. The effect of GHKGD on the **(B)** lung index and **(C)** lung wet/dry weight of mice was assessed (n = 6). Inhibition of NP expression at the mRNA and protein levels by GHKGD in mice following influenza virus infection was detected: **(D)** mRNA level; **(E)** protein level. **(F)** Plaque reduction assay. MDCK cells were infected with mouse lung tissue supernatant, and the infected cells were then cultured and overlaid with MEM supplemented with 1% low melting point agarose and 2.5 μg/mL TPCK‐treated trypsin. The number of plaques was calculated. The data are presented as the means ± SDs of the results from three independent experiments, and were analyzed by ANOVA. #*p* < 0.05, ##*p* < 0.01 compared to the normal control group, *p < 0.05, **p < 0.01 compared to the model group. GHKG‐L, Ganghuo Kanggan Decoction with low dose; GHKG‐H, Ganghuo Kanggan Decoction with high dose.

Similarly, the lungs of infected mice were harvested on day 5 post-infection, and pathological changes were assessed (Figure 6). As shown in Figure 5B, extensive bronchial epithelial cell necrosis, alveolar damage, and significant cellular infiltration were observed in the lungs of mice in the model group. In contrast, in mice treated with GHKGD, lung hyperaemia was decreased and histopathological changes were reduced in a dose-dependent manner (Figures 5D,E). These results indicated that GHKGD ameliorated lung damage in virus-infected mice.

### Effects of Ganghuo Kanggan Decoction on the RIG-I-like Receptors Pathway in H1N1 IAV-Infected Mice

**FIGURE 7:**
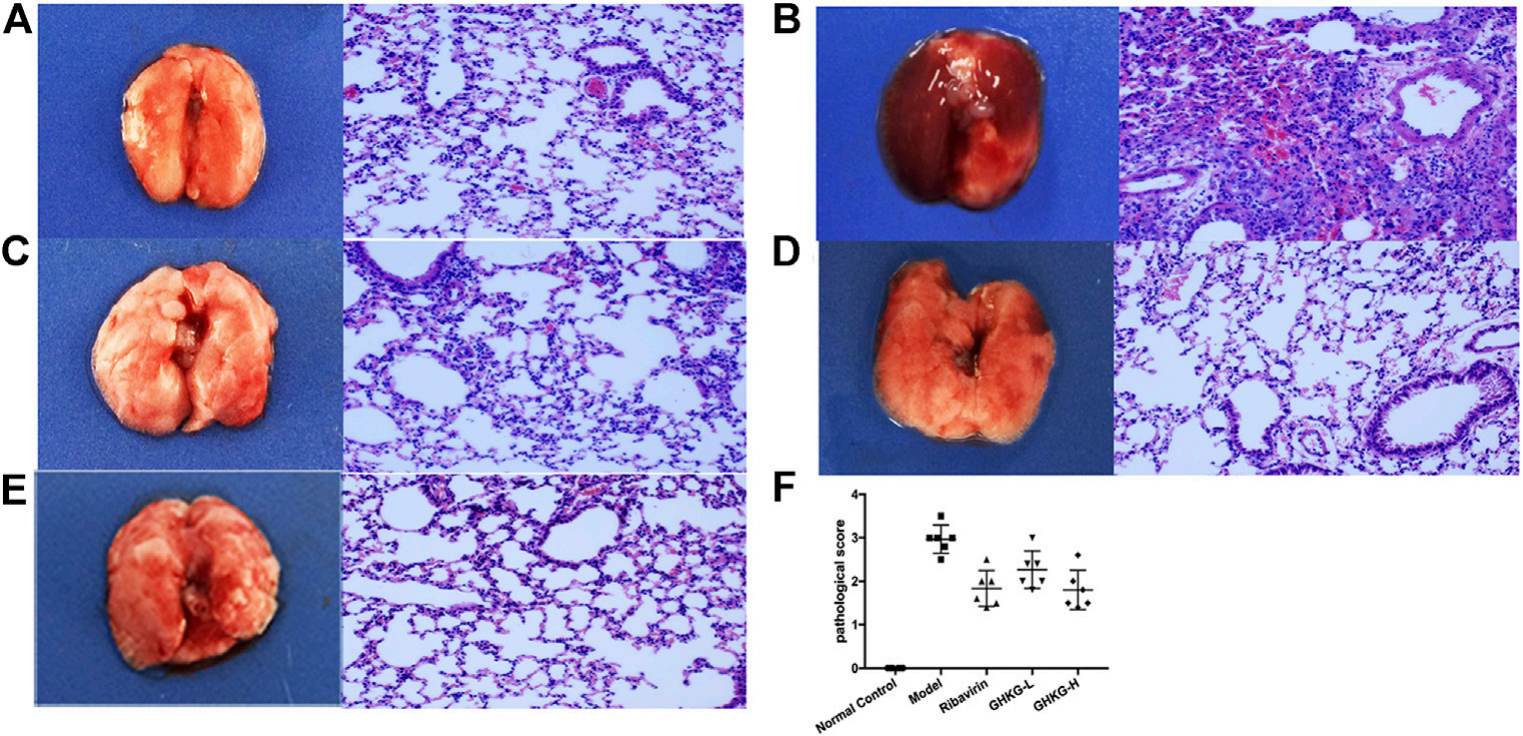
Histological observations of lung tissues formice sacrificed at the fifth d.p.i. (HE, × 200) (n = 6) **(A)** Mock‐infected mice treated with PBS (normal control, NC); **(B)** IAV-infected mice treated with PBS (viral control); **(C)** IAV‐infected mice treated with ribavirin (75 mg/kg/d); **(D‐E)** IAV‐infected mice treated with GHKGD (46.2 and 11.6 g/kg/day, respectively); **(F)** pathological scores. The data are presented as the means ± SDs (n = 6) and were analyzed by ANOVA. #*p* < 0.05, ##*p* < 0.01 compared to the normal control group, *p < 0.05, **p < 0.01 compared to the model group. GHKG‐L, Ganghuo Kanggan Decoction with low dose; GHKG‐H, Ganghuo Kanggan Decoction with high dose.

The RLR signaling pathway is a well-known innate immune pathway (Chen et al., 2018). As a member of the RIG-I-like receptor (RLR) family, retinoic acid-induced gene I (RIG-I) can recognize influenza virus RNA, activate the production of type I interferon (IFNs) and regulate the corresponding immune responses (Iwasaki and Pillai, 2014; Long et al., 2019; Yoneyama et al., 2015). Therefore, we measured the levels of IFN-α and IFN-β in mouse serum (Figures 7A,B). The results showed that the production of IFN-α and IFN-β was inhibited by GHKGD. In addition, the expression of RIG-I was measured at the mRNA and protein levels in mouse lung tissues. The expression of RIG-I was markedly increased in the model group compared with the normal control group (Figures 7C,D). Ribavirin and GHKGD inhibited RIG-1 expression at the mRNA and protein levels compared with that in the model group. In addition, the expression of mitochondrial antiviral-signaling protein (MAVS), interferon regulatory factor 3 (IRF3) and interferon regulatory factor 7 (IRF7) was inhibited by GHKGD (Figure 7E). The nuclear factor kappa-B (NF- κB) pathway, which is important for the regulation of inflammation and apoptosis, is one of the signal cascades induced by influenza virus infection (Ding et al., 2017; Yan et al., 2018). As shown in Figure 7F, compared to that in the normal control group, the expression of NF-κB p65 was significantly increased in the model group. Ribavirin and GHKGD markedly reduced the level of NF-κB p65 after IAV infection compared with that in the model group. STAT1 also participates in the regulation of various inflammatory mediators, and plays an important role in the production of pro-inflammatory cytokines (Meraz et al., 1996). Our results showed that the STAT1 expression was inhibited by GHKGD, thereby avoiding the excessive secretion of inflammatory factors (Figure 7F). These results indicated that GHKGD downregulated the expression of RIG-1, MAVS, IRF3, IRF7, NF-κB p65 and STAT1 in the RLR signaling pathway, thereby inhibiting excessive inflammatory responses.

### Effects of Ganghuo Kanggan Decoction on the Levels of TH1 and TH2 Cytokines in H1N1 IAV-Infected Mice

**FIGURE 8:**
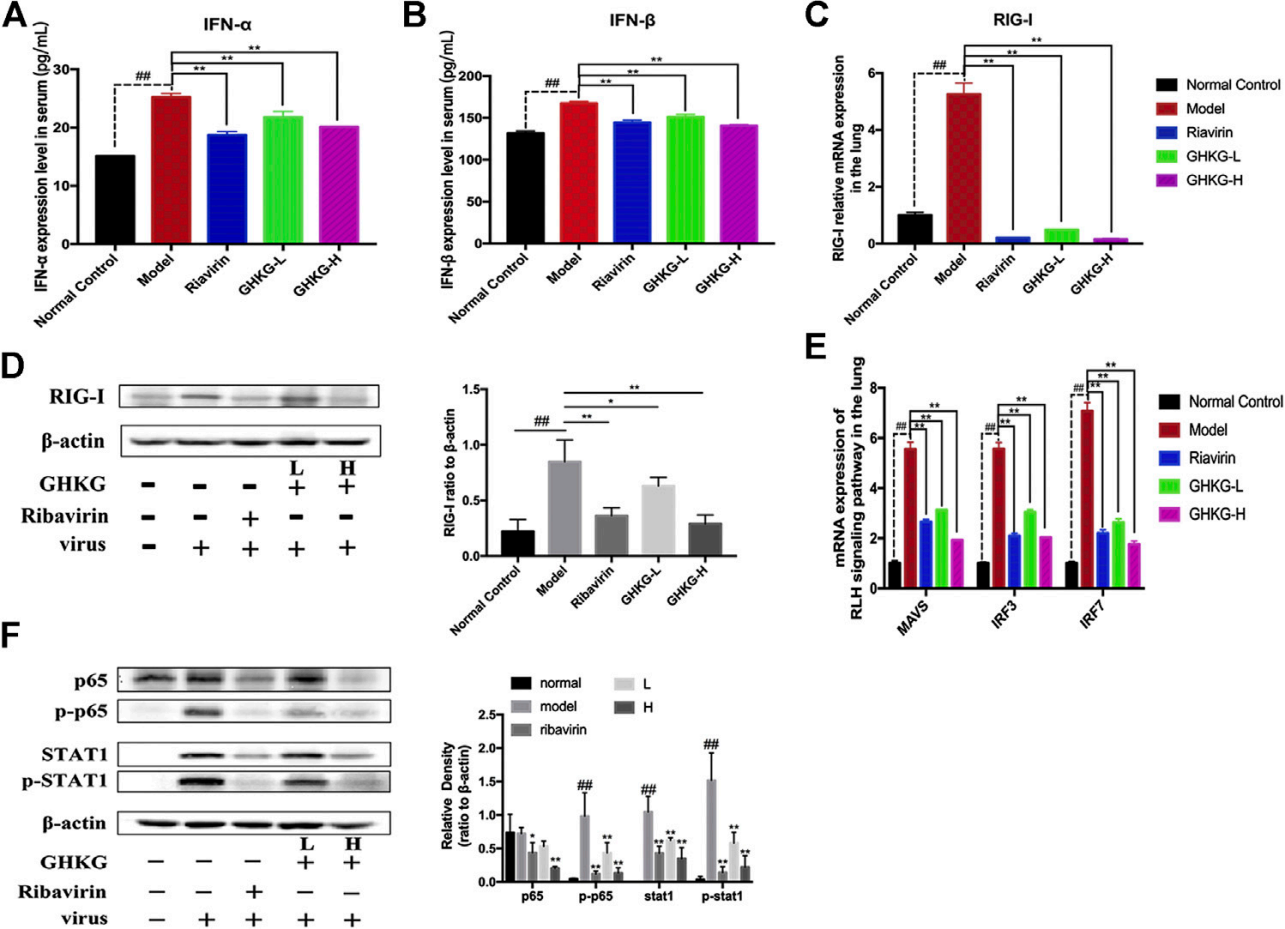
Inhibitory effect of GHKGD on IFN‐α and IFN‐β levels in serum in mice following influenza virus infection (n = 6). **(A)** IFN‐α level; **(B)** IFN‐β level. Inhibition of RIG‐1 expression at the mRNA and protein levels by GHKGD in mice following influenza virus infection. **(C)** mRNA level; **(D)** protein level. Mice were infected with influenza A/Puerto Rico/8/1934(H1N1) virus (2 LD 50 ) and treated with GHKGD (46.2 and 11.6 g/kg/day, respectively) or ribavirin (70mg/kg/d) once a day for 5 days. The expression of RIG‐1 was measured by RT‐PCR and Western blot assays. **(E)** Relative mRNA expression levels of MAVS, IRF3, and IRF7 in the RLR signaling pathway. The data are presented as the means ± standard deviations of the results from three independent experiments. **(F)** Relative protein expression levels of p65, p‐p65, STAT1, p‐STAT1 and β‐actin in the RLR signaling pathway. The data are presented as the means ± SDs of the results from three independent experiments, and were analyzed by ANOVA. #*p* < 0.05, ## *p* < 0.01 compared to the normal control group, *p< 0.05, **p < 0.01 compared to the model group. GHKG‐L, Ganghuo Kanggan Decoction with low dose; GHKG‐H, Ganghuo Kanggan Decoction with high dose.

The serum levels of Th1 (IL-2, TNF-α, and IFN-γ) and Th2 (IL-4 and IL-5) cytokines were tested by a flow cytometer. The results showed that, compared with the normal control group, the model group exhibited higher levels of TNF-α, IL-2, and IFN-γ cytokines, while the changes in the levels of IL-5 and IL-4 were not statistically significant. In addition, Ribavirin and GHKGD groups exhibited lower levels of IL-2, TNF-α, and IFN-γ cytokines compared with that in the model group (Figures 8A–C). The IL-5 and IL-4 levels were markedly increased in GHKGD group, while the Ribavirin group changed without statistically significant (Figures 8D,E). These findings suggest that the differentiation of Th1 and Th2 cytokines exert critical functions during the occurrence of influenza virus pneumonia, and the GHKGD could played an immunomodulatory role by downgrading Th1 (IL-2, TNF-α, and IFN-γ) cytokines, while raising Th2 (IL-4 and IL-5) cytokines.

**FIGURE 9:**
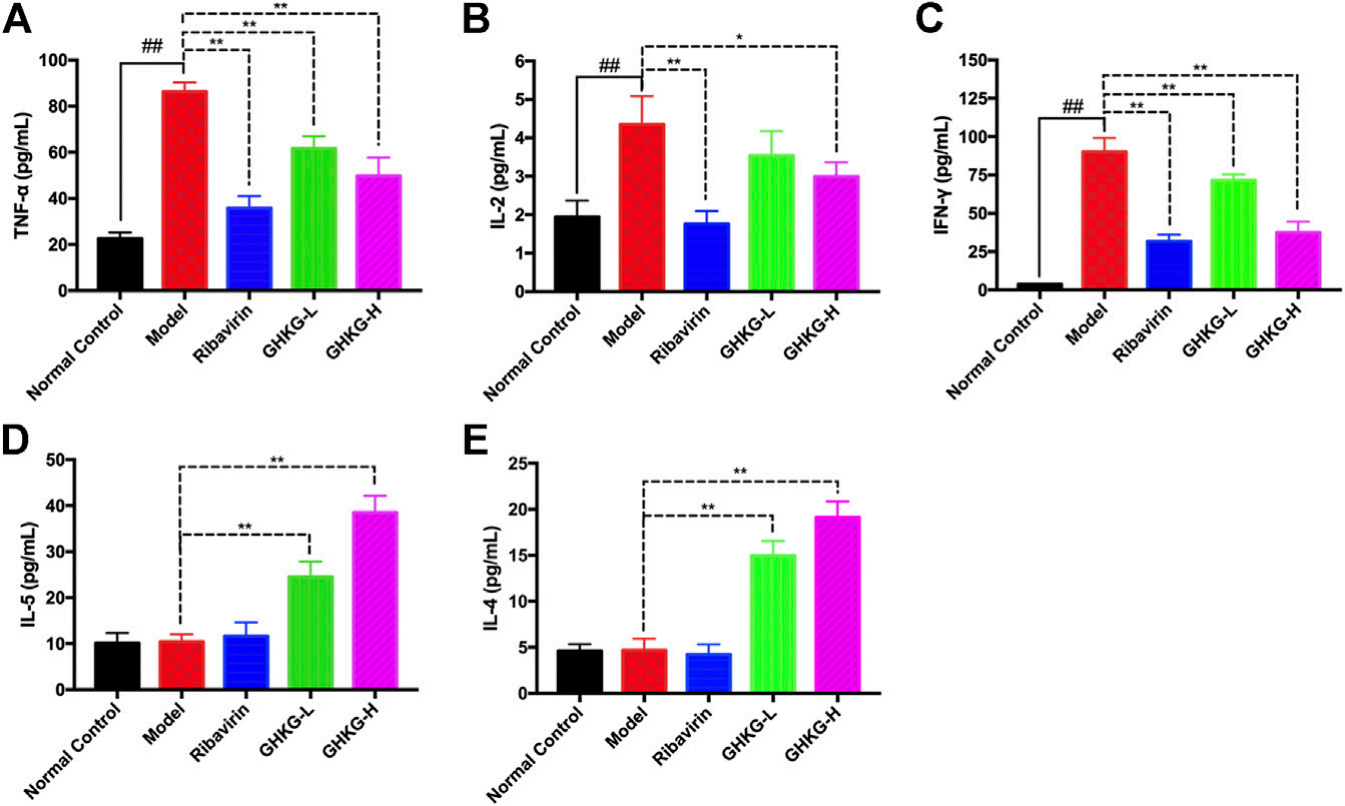
Effect of the GHKGD on serum cytokine levels. The serum levels of TNF-α **(A)**, IL-2 **(B)**, IFN-γ **(C)**, IL-5 **(D)**, and IL-4 **(E)** were detected by BD cytometric bead array (CBA) mouse Th1/Th2 cytokine kit. The data are presented as the means ± SDs of the results from three independent experiments, and were analyzed by ANOVA. ^#^P < 0.05, ^##^P < 0.01 compared to the normal control group, *P < 0.05, **P < 0.01 compared to the model group. GHKG-L, Ganghuo Kanggan Decoction with low dose; GHKG-H, Ganghuo Kanggan Decoction with high dose.

## Discussion

ARDS and respiratory failure are the main causes of death from influenza virus infection. Drug resistance and adverse reactions to antiviral drugs have prompted scientists to consider searching for host target-based drugs to reduce viral damage to the respiratory system. Many TCMs have been used to treat influenza, and TCMs may be a potential source of alternative compounds for drug design and discovery. GHKGD is a clinical experience prescription used for the treatment of viral pneumonia in the Lingnan area (Zhao et al., 2018). In our previous clinical study, GHKGD was found to be not only effective but also safe. Compared with oseltamivir, Ganghuo Kanggan decoction shortened the antipyretic time of patients, improved their cold symptoms, inhibited the dominant response of influenza virus Th1 cell subsets, reversed the Th1/Th2 imbalance, and reduced immunoinflammatory damage (Yang et al., 2017b). The combination of oseltamivir and Ganghuo Kanggan decoction had a more obvious clinical effect than either alone (Lyv et al., 2017). In addition, *in vivo* studies have shown that GHKGD could significantly reduce the exudation of inflammatory mediators from influenza virus-induced viral pneumonia in mice and improve the survival rate of mice (Chen et al., 2015; Liu et al., 2015). Based on this, we adopted the strategy of network pharmacology (Xu et al., 2017) to predicted its possible pharmacological mechanism, and carried out *in vivo* experiments to confirm it.

Through a network pharmacology approach, we identified 116 potential active components and 17 targets. Some of the main active ingredients have been reported to have direct antiviral effects or to reduce pneumonia by suppressing inflammation; for example, isoliquiritigenin reduced influenza virus-induced lung inflammation and mortality in mice (Traboulsi et al., 2015), luteolin suppressed coat protein I complex expression to decrease the yield of IAV *in vitro* (Yan et al., 2019), and kaempferol exhibited a protective effect on H9N2 virus-induced inflammation via the suppression of the TLR4/MyD88-mediated NF-κB and MAPK pathways (Zhang et al., 2017). These results show that the strategy of applying network pharmacology to find potential active compounds is reliable and feasible. Therefore, in future work, we will further study whether other potential active ingredients have direct antiviral or anti-inflammatory effects.

Enrichment analysis of the 17 potential targets showed that GHKGD mainly regulated type I interferon-related signaling pathways to play an anti-influenza role in the body. These findings suggested that GHKGD may prevent the overexpression of inflammatory mediators in IAV-infected BALB/c mice by inhibiting the RLR signaling pathway. Then we designed *in vivo* experiments to confirm this hypothesis.

First, it can be seen that, in the BALB/c mouse model, on the fifth day after IAV infection, the body weight of the mice decreased, the lung index and lung wet/dry weight ratio increased significantly, and extensive bronchial epithelial cell necrosis, alveolar injury and obvious cellular infiltration were observed in lung tissues. However, the ribavirin group and GHKGD groups (high and low dose) of mice exhibited a significant improvement in the lung index and lung wet/dry weight ratio, reductions in NP protein expression in lung tissue and virus titers in the lung, and amelioration of lung tissue injury. These results indicated that GHKGD was an effective oral drug to control the inflammation caused by influenza A virus infection.

Second, we investigated whether the antiviral role of GHKGD involves the RLR signaling pathway in the lungs. After influenza virus infects the host, RIG-I, a member of the RIG-I-like receptor (RLR) family, can recognize intracellular viral RNA, leading to the activation of interferon type I and the entry of NF-κB and STAT1 into the nucleus and promoting the production of pro-inflammatory factors and pro-inflammatory chemokines (Zhu et al., 2014). An excessive immune response is considered to be a predictor of influenza-mediated death (Nimmerjahn et al., 2004). In this study, the expression of RIG-I, NF-κB and STAT1 proteins, the level of MAVS and IRF3/7 mRNA and the content of IFN-α/β in lung tissue of the model group were significantly increased, indicating that the RIG-I/NF-κB/STAT1 signaling pathway was activated. After oral administration of GHKGD to mice, pulmonary inflammation was alleviated, RIG-I, NF-κB and STAT1 protein overexpression was inhibited, and the content of IFN-α/β was decreased. These effects suggested that GHKGD might inhibit excessive inflammatory reactions by regulating the RLR signaling pathway.

It is well known that, influenza virus, a strict intracellular pathogenic microorganism, mainly stimulates Th cells to differentiate into Th1 cells and releases their signature cytokines (IFN-γ, TNF-α, IL-2) after infecting the host, while the differentiation of Th2 cells and their cytokine expression (IL-4, IL-5) were obviously suppressed (Yan et al., 2018). The balance between pro-inflammatory factors and anti-inflammatory factors mediates the process of immune damage and immune regulation after influenza virus infection. In the present study, we investigated the effect of GHKGD on Th1/Th2 imbalance in IAV-infected mice. The results demonstrated that GHKGD could significantly increase the expression of Th1 ((IL-2, TNF-α, and IFN-γ)) cytokines, and reduced the secretion of Th2 (IL-5 and IL4) cytokines, which was crucial for reducing the inflammatory factors overexpression and avoiding the production of inflammatory factor storms.

## Conclusion

In conclusion, the network pharmacological analysis of GHKGD identified 116 compounds and 17 target genes associated with influenza virus infection. EGFR, JAK3, PTPN1, JAK2, ISG20, PTPN2, STAT3, PTPN11 and STAT1 were recognized as hub genes. According to the results of pathway enrichment analysis, we verified the protective effect of GHKGD against pneumonia in mice infected with IAV and determined that GHKGD can prevent excessive inflammation by downregulating the RLR signaling pathway and regulating the balance of Th1/Th2. Our findings confirmed that network pharmacology strategies can match candidate compounds with potential targets via the construction of multi-component networks. These findings provide both a new option for the treatment of IAV infection and information to further reveal the mechanisms of GHKGD.

## Data Availability Statement

The original contributions presented in the study are included in the article/Supplementary Material, further inquiries can be directed to the corresponding authors.

## Author Contributions

YaL, SZ, YJ, GL, and XL conceived and designed the experiments. YaL, QZ, HL, TH, and ZL participated in the specific experimental process. YaL, QZ, HL, and ZL performed the research and analyzed the data. YaL wrote the paper. YaL, GL, YiL, and XL drafted the manuscript. YaL, YJ, SZ, and XL revised the manuscript. All authors approved and agreed to be responsible for all aspects of the work.

## Funding

This work was supported by the National Natural Science Foundation of China (Grant No. 81973814) and Key-Area Research and Development Program of Guangdong Province (Grant No. 2020B1111100002).

## Conflict of Interest

The authors declare that the research was conducted in the absence of any commercial or financial relationships that could be construed as a potential conflict of interest.
